# Discovery of *Rickettsia* spp. in mosquitoes collected in Georgia by metagenomics analysis and molecular characterization

**DOI:** 10.3389/fmicb.2022.961090

**Published:** 2022-09-08

**Authors:** Adam R. Pollio, Ju Jiang, Sam S. Lee, Jaykumar S. Gandhi, Brian D. Knott, Tamar Chunashvili, Matthew A. Conte, Shannon D. Walls, Christine E. Hulseberg, Christina M. Farris, Drew D. Reinbold-Wasson, Jun Hang

**Affiliations:** ^1^Walter Reed Army Institute of Research, Silver Spring, MD, United States; ^2^Naval Medical Research Center, Silver Spring, MD, United States; ^3^Henry M. Jackson Foundation for the Advancement of Military Medicine, Inc., Bethesda, MD, United States; ^4^U.S. Army Medical Research Directorate - Georgia (USAMRD-G), Walter Reed Army Institute of Research, Tbilisi, Georgia

**Keywords:** pathogen discovery, *Rickettsia*, NGS, vector-borne disease surveillance, country of Georgia, One Health, metagenomic, mosquito

## Abstract

Arthropods have a broad and expanding worldwide presence and can transmit a variety of viral, bacterial, and parasite pathogens. A number of *Rickettsia* and *Orientia* species associated with ticks, fleas, lice, and mites have been detected in, or isolated from, patients with febrile illness and/or animal reservoirs throughout the world. Mosquitoes are not currently considered vectors for *Rickettsia* spp. pathogens to humans or to animals. In this study, we conducted a random metagenome next-generation sequencing (NGS) of 475 pools of *Aedes*, *Culex*, and *Culiseta* species of mosquitoes collected in Georgia from 2018 to 2019, identifying rickettsial gene sequences in 33 pools of mosquitoes. We further confirmed the findings of the *Rickettsia* by genus-specific quantitative PCR (qPCR) and multi-locus sequence typing (MLST). The NGS and MLST results indicate that *Rickettsia* spp. are closely related to *Rickettsia bellii*, which is not known to be pathogenic in humans. The results, together with other reports of Rickettsia spp. in mosquitoes and the susceptibility and transmissibility experiments, suggest that mosquitoes may play a role in the transmission cycle of *Rickettsia* spp.

## Introduction

Arthropod vectors, such as mosquitoes, ticks, fleas, flies, lice, and midges, have a broad and expanding worldwide presence and can transmit a variety of viral, bacterial, and parasite pathogens between animals and humans. Vector-borne diseases (VBDs) result in millions of cases of illness and hundreds of thousands of deaths each year (Molyneux, 1998; Schmidt et al., 2013; Huntington et al., 2016; Abdad et al., 2018). VBDs pose enormous social and economic burdens on the world, particularly on those living in low-income countries and rural areas, where the public health resources are insufficient for effective transmission prevention and medical treatment (Eisen et al., 2017; Wilcox et al., 2019). In addition, the impact of climate change is increasingly affecting developed countries due to the expanded mosquito and tick habitats in these warming areas (Semenza and Suk, 2018; Wilcox et al., 2019). These interrelated issues have increased VBDs to one of the top concerns of the One Health program, a global and multidisciplinary effort to confront health issues that arise from complex environmental, agricultural, and human factors (Schmidt et al., 2013; Sánchez-Montes et al., 2021). VBDs by different pathogens or strains of a pathogen can lead to highly variable clinical symptoms ranging from mild to severe to even fatal. The systematic and in-depth surveillance of vectors, vector-borne pathogens, and genetic evolution of pathogens has become essential for the prevention and control of VBDs and prompt outbreak response (Gillespie et al., 2008; Schmidt et al., 2013; Vayssier-Taussat et al., 2015; Chala and Hamde, 2021).

Rickettsial diseases are caused by infection with members of the genera *Rickettsia* and *Orientia*, the family *Rickettsiaceae*. The agents responsible for these infections include spotted fever group rickettsiae (SFGR), typhus group rickettsiae (TGR), scrub typhus group orientia, and transition group rickettsiae (Hechemy et al., 2003; Abdad et al., 2018). Additionally, there is an ancestral group of rickettsiae, which diverged genetically earlier than the aforementioned groups and includes agents, such as *Rickettsia bellii*, which are not known to cause disease in humans (Stothard et al., 1994; Weinert et al., 2009). A number of *Rickettsia* and *Orientia* species associated with ticks, fleas, lice, and mites have been detected in, or isolated from, patients with febrile illness and/or animal reservoirs throughout the world (Blair et al., 2004; Abdad et al., 2018). Mosquitoes are not currently considered vectors for *Rickettsia* spp. pathogens. However, there are an increasing number of reports of *Rickettsia* spp. in mosquitoes, and, importantly, a recent study by Dieme C. et al. demonstrated the ability of *Anopheles gambiae* mosquitoes to act as a vector and transmit *R. felis* to mice in a laboratory setting (Socolovschi et al., 2012a,b; Dieme et al., 2015; Guo et al., 2016; Maina et al., 2017; Barua et al., 2020). More studies are needed to identify *Rickettsia* spp. in mosquitoes, characterize mosquito-borne rickettsiae, assess the role of mosquitoes in the transmission cycle of rickettsiae to humans or animals, and ultimately find evidence for human infections.

In this study, we conducted a random metagenome next-generation sequencing (NGS) of *Aedes*, *Culex*, and *Culiseta* mosquitoes captured in the country of Georgia to identify rickettsial gene sequences in mosquito pools. The findings were confirmed by *Rickettsia* genus-specific quantitative PCR (qPCR) and multi-locus sequence typing (MLST). The results support our previous report on mosquito-borne rickettsia in the Republic of Korea (ROK), suggesting a potential role for mosquitoes in the transmission cycle of *Rickettsia* spp.

## Materials and methods

### Mosquito collection

Mosquitoes were collected in Georgia throughout the 2018 and 2019 surveillance seasons (April–October). Multiple collection methods were utilized to survey a diversity of mosquito species including battery powered mosquito traps—BG Sentinel 2 (Biogents AG, Regensburg, Germany), a CDC light trap (Model 1012 and 1212, John W. Hock Company, Gainesville, FL, United States), a Stealth Trap (Model 214, John W. Hock Company, Gainesville, FL, United States), and a Fay-Prince Trap (Model 812, John W. Company, Gainesville, FL, United States); mosquito aspiration, mouth aspirators (Model 612, John W. Hock Company, Gainesville, FL, United States), and an Improved Prokopack aspirator (Model 1419, John W. Hock Company, Gainesville, FL, United States); mosquito larval collection—a mosquito dipper (Model 320, John W. Hock Company, Gainesville, FL, United States). Powered traps were equipped with mosquito attractants during surveillance: dry ice (1 KG/trap/24 h) in insulated dry ice containers (John W. Hock Company, Gainesville, FL, United States) utilized in a majority of the collections; BG-Lure (Biogents AG, Regensburg, Germany) with the BG Sentinel 2 traps; CDC-LT and Fay-Prince traps used either incandescent and ultraviolet (UV) light sources in addition to CO_2_. Powered traps were set to work for 24 h collection periods and all trapped and aspirated specimens were transported frozen for storage and processing. Collected larval mosquitoes were placed in sealed containers until emergence, then frozen and processed with the other specimens. Mosquitoes were morphologically identified using a stereomicroscope (Leica S4E, Leica microsystems, Germany) and the ECDC MosKey Tool.^1^ Female specimens were sorted by species into pools not greater than 30 individuals and stored at −80^°^C. A subset of the total collection was shipped frozen from Tbilisi, Georgia to Walter Reed Army Institute of Research (WRAIR), Silver Spring, Maryland, United States.

### Mosquito extraction and nucleic acids purification for metagenome sequencing

Mosquito pools were homogenized in the cell culture medium by bead beating using a Mini-Beadbeater-16 (BioSpec Products, Inc., Bartlesvelle, OK, United States) and centrifuged to harvest clear supernatant, as described previously (Sanborn et al., 2019, 2021). After pre-treatment of incubation with nucleases to reduce host DNA and RNA contents, clear supernatant was extracted to purify nucleic acids using the MagMAX Pathogen RNA/DNA Kit and the KingFisher Flex Purification System (Thermo Fisher Scientific) by following the manufacturer’s user guides.

### Metagenome sequencing

Purified nucleic acid samples were subjected to unbiased random reverse transcription and PCR amplification (RT-PCR), as described previously (Maina et al., 2017; Sanborn et al., 2019, 2021). Separate reactions of negative control (molecular biology grade water) and positive control (MS2 bacteriophage RNA) were included as quality control to estimate background noise in the lab and to track cross contaminations. Random RT-PCR amplicons were purified, examined using the TapeStation System and D5000 ScreenTape (Agilent Technologies, Inc., Santa Clara, CA, United States) or using the Quant-iT PicoGreen dsDNA Assay (Thermo Fisher Scientific), subjected to library preparation using the Illumina DNA Prep Kit, followed by NGS using the MiSeq System and Reagent Kit v3 (600-cycle) (Illumina, San Diego, CA, United States).

### Metagenomics data analyses

Next-generation sequencing data were processed and analyzed using a metagenomics analysis pipeline, which includes sequence read quality processing, host sequence removal, *de novo* sequence data assembly and contig scaffolding, megablast, and discontiguous megablast of the contigs and unassembled single reads, and the iteration of sequence-based taxonomic entities in the specimens (Kilianski et al., 2015). Assembled sequences from the pipeline were curated using bioinformatics tools, an NGS_Mapper, Geneious R10 (Biomatters Ltd.),^2^ an Integrative Genomics Viewer (IGV) (Broad Institute),^3^ for GenBank submission and phylogenetic analyses. The MUltiple Sequence Comparison by Log-Expectation (MUSCLE) program was used for multiple sequence alignment. A maximum likelihood phylogenetic tree was generated using IQ-TREE version 1.6.12 using these alignments with 1,000 standard non-parametric bootstrap replicates specified (-b 1,000) (Minh et al., 2020). Output trees were visualized and adapted for figures using FigTree version v1.4.4.^4^

*De novo* clustering of the metagenome sequences was performed using QIIME2 command line version 2022.2 (Bolyen et al., 2019). The “vsearch dereplicate-sequences” command was used first, followed by the “vsearch cluster-features-*de-novo*” command using a percentage identity (–*p*) of 0.99. Abundance counts are provided for each Operational Taxonomic Unit (OTU). Principal component analysis (PCA) was calculated on this OTU abundance output using the python scikit-learn decomposition package and plotted.

### Mosquito DNA extraction, rickettsial real-time PCR assay, and amplicon sequencing

DNA was extracted from 100 μl of mosquito homogenates using the DNeasy blood & tissue kit (QIAGEN), along with one negative control, and the purified DNA was eluted in the 50 μl of elution buffer. Then, a 1:10 diluted stock was prepared for use in Rickettsia genus-specific qPCR assays.

Two qPCR assays were attempted: the Rick17b assay targets the 17 KDa antigen gene (Jiang et al., 2012) and the RickCS assay targets the citrate synthase gene (*glt*A) (Stenos et al., 2005). The reaction final conditions and the cycling parameters were the same as reported previously (Jiang et al., 2012).

PCR was performed using primers targeting the 16S, 23S, *gltA*, *ompA*, *ompB*, and *sca4* genes (Table 1) (Fournier et al., 1998; Roux and Raoult, 2000; Jiang et al., 2005, 2012, 2013; Guo et al., 2016; Maina et al., 2017). Additional primers for the 23S rRNA gene and *ampB* gene were designed targeting the conserved sequences of *Rickettsia*, or *Rickettsia* and *Orientia* genus based on the sequence alignment using MEGA 11 with 20 *Rickettsia* species and 5 *O. tsutsugamushi* strains. Primers for *ompB* in this study were designed specifically targeting *R. bellii* (GenBank CP000087). The 25 μl reaction mixture contained 2 μl of purified DNA, the Phusion Flash High-Fidelity PCR Master Mix (Thermo Fisher Scientific), and 0.3 μM of forward and reverse primers. PCR reactions were conducted on a T-Gradient Thermocycler (Biometra, Göttingen, Germany), incubated at 98°C for 10 s followed by 35 cycles of denaturation at 98°C for 1 s, annealing at 51–61°C (based on the Tm of the primers calculated on the website^5^) for 5 s, and elongation at 72°C for 30 s. Following the completion of the amplification steps, the reaction mixtures were exposed to a final elongation step at 72°C for 2 min. PCR products were visualized with GelRed on 1.0% agarose gels following electrophoresis. The mastermix for PCR was prepared in a clean hood separated from where the DNA templates were added, and negative control (molecular biology grade water) was run at the same time under the same condition as the samples. The genomic DNA of *Rickettsia africae* was used as a positive control.

**TABLE 1:** Primers used for PCR, nested PCR, and Sanger sequencing.

Gene	Primer	Sequence (5′-3′)	References
*rrs*	16SU17F	AGAGTTTGATCCTGGCTCAG	Jiang et al., 2012
	16SOR1198R	TTCCTATAGTTCCCGGCATT	Maina et al., 2017
	16SU547F	CAGCAGCCGCGGTAATAC	Maina et al., 2017
	16SU833R	CTACCAGGGTATCTAATCCTGTT	Maina et al., 2017
23S	23SU14F	AAGAGCATTTGGTGGATG	This study
	23SR2753R	AATCAATCGAGCTATTAGTATC	This study
	23SOR655F	TGAATTAGACCCGAAACCG	This study
	23SU1240F	TCGGAAGTGAGAATGCT	This study
	23SOR1897F	GTGAAGATGCGGAGTTC	This study
	23SR722R	CCTTCAGCGGATTTTACTC	This study
	23SOR1371R	TACGCCTTTCAGCCTCA	This study
	23SU2054R	CAAAAGGGTGGTATCTCAA	This study
*gltA*	CS151F	CCGGGYTTTATGTCTACTGC	Guo et al., 2016
	CS1259R	AGCTGTCTWGGTCTGCTGATT	Guo et al., 2016
*ompA*	190-70F	ATGGCGAATATTTCTCCAAAA	Fournier et al., 1998
	RompA642R	ATTACCTATTGTTCCGTTAATGGCA	Jiang et al., 2012; Minh et al., 2020
	190-701R	GTTCCGTTAATGGCAGCATCT	Fournier et al., 1998
	RompA58F	GGAGTAHKTTAGAKTTTAACGG	Jiang et al., 2005
	RompA657R	TATTTGCATCAATCSYATAAGWA	Jiang et al., 2005
*ompB*	120-M59F	CCGCAGGGTTGGTAACTGC	Roux and Raoult, 2000
	120-807R	CCTTTTAGATTACCGCCTAA	Roux and Raoult, 2000
	ompB1570R	TCGCCGGTAATTRTAGCACT	Jiang et al., 2013
	RBelB-1F	ATGATGATGAATGAAGCCTCTAAT	This study
	RBelB-28F	ATTTTAGGACCTAATGGTGTT	This study
	RbelB2742R	CTAGAAGTTTAGGCGGACT	This study
	RbelB1427R	TCACCTTGGATTAAAGTATAGG	This study
	RbelB1283F	CTTTGACATCAGATGAAGTTATG	This study
*sca4*	RrD749F	TGGTAGCATTAAAAGCTGATGG	Jiang et al., 2005
	RrD928F	ATTTATACACTTGCGGTAACAC	Jiang et al., 2005
	RrD1826R	TCTAAATKCTGCTGMATCAAT	Jiang et al., 2005
	RrD2685R	TTCAGTAGAAGATTTAGTACCAAAT	Jiang et al., 2005

For Sanger sequencing, PCR amplicons were purified using the QIAquick PCR purification kit (QIAGEN). Sequencing reactions were performed for both DNA strands using the Big Dye Terminator v3.1 Ready Reaction Cycle Sequencing Kit (Thermo Fisher Scientific Applied Biosystems, Foster City, CA). After purification of sequenced products using Performa DTR Gel Filtration Cartridges (Calibre Scientific EdgeBio, Holland, OH, United States), Sanger sequencing was run on a 3500 Genetic Analyzer (Applied Biosystems). The primers used for PCR amplification were also used for the sequencing reactions. Sequences were assembled using the CodonCode aligner (CodonCode Corporation, Barnstable, MA, United States).

The nucleotide sequences from this study were deposited in GenBank with accession numbers ON960050-ON960051 for *gltA* genes, OP007139-OP007153 for 16S rRNA genes, and OP007301-OP007317 for 23S rRNA genes.

## Results

**FIGURE 1:**
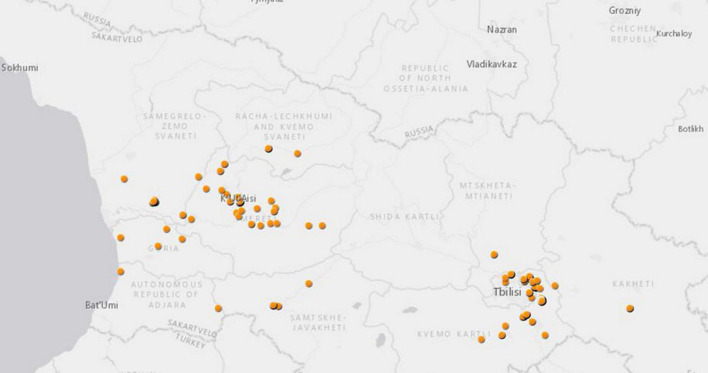
Mosquito collection in Georgia from 2018 to 2019 for this study. Each dot represents GPS coordinates of each mosquito collection site.

In total, 475 pools (5,146 specimens) of *Aedes*, *Culex*, *Culiseta* mosquitoes were studied in this metagenomics analysis. The mosquitoes were collected at 112 Global Positioning System (GPS) locations in 10 provinces across Georgia from 2018 to 2019 (Table 2 and Figure 1). Many mosquitoes were from two Georgian military training bases, Krtsanisi training area (KTA, 177 pools) and the Norio training area (NTA, 36 pools), both located in the southeastern province of Kvemo Kartli.

**TABLE 2:** Summary of mosquitoes collected across Georgia from 2018 to 2019 utilized in this study.

Mosquito species by site	Total number of mosquitoes (pools)	Number of pools *Rickettsia* positive
**Training base Krtsanisi^a^**		
*Aedes albopictus*	1 (1)	0
*Aedes caspius*	930 (63)	2^a^
*Aedes surcofi*	34 (5)	1^a^
*Aedes vexans*	51 (6)	0
*Culex pipiens*	1037 (71)	25^a^
*Culex pusillus*	156 (8)	3^a^
*Culex theileri*	17 (4)	0
*Culex tritaeniorhynchus*	253 (20)	1^a^
**Training base Kutaisi^a^**		
*Aedes albopictus*	3 (2)	0
*Culex pipiens*	119 (13)	0
**Training base Norio^a,b^**		
*Aedes albopictus*	5 (2)	1^b^
*Aedes geniculatus*	45 (7)	0
*Aedes vexans*	119 (9)	0
*Culex pipiens*	145 (17)	0
*Culex pusillus*	1 (1)	0
**Training base Senaki and Camp Eki^a^**		
*Aedes albopictus*	35 (6)	0
*Culex pipiens*	568 (51)	0
**Training base Vaziani^a^**		
*Aedes albopictus*	5 (4)	0
*Aedes caspius*	55 (17)	0
*Aedes surcofi*	12 (1)	0
*Culex pipiens*	542 (60)	0
*Culex theileri*	43 (6)	0
**Other sites within Georgia^b,c^**		
*Aedes albopictus*	182 (48)	0
*Aedes caspius*	2 (2)	0
*Aedes vexans*	40 (4)	0
*Culex pipiens*	919 (71)	0
*Culex pusillus*	28 (2)	0
*Culex theileri*	20 (2)	0
*Culiseta longiareolata*	51 (4)	0

Collection methods: ^a^Powered traps.

^b^Larval dipping.

^c^Aspiration.

**FIGURE 2:**
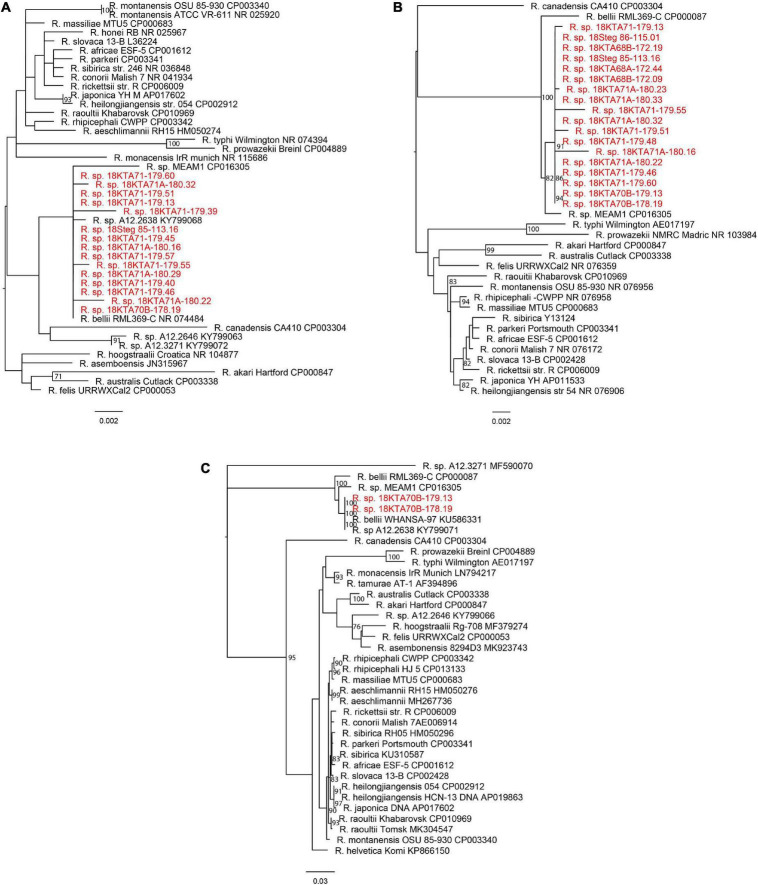
Phylogeny of *Rickettsia* sp. from this study and known rickettsial strains. **(A)** 16S rRNA gene; **(B)** 23S rRNA gene; and **(C)** *gltA* gene. The maximum likelihood phylogenetic tree with bootstrap support values from 1,000 replicates is shown at the branches. The scale bar represents estimated nucleotide substitutions per site. The sequences from this study are shown in red.

The unbiased metagenomics approach identified a large variety of viruses and other microbes associated with these pools of mosquitoes. *Rickettsia* spp. sequences were found in 33 mosquito pools, with each pool having over 500 rickettsial reads and/or assembled contig(s) of 500 bp or longer in sequence length (Table 3). As expected, only 23S and 16S ribosomal RNA genes were identified by metagenome sequencing, in which the abundant ribosome RNA transcripts were efficiently amplified and sequenced. Both Genbank nucleotide BLAST analysis (Table 3) and phylogenetic analysis (Figure 2) clearly indicated that Georgian mosquito-associated rickettsiae are closely related to *R. bellii*. The phylogeny also suggested that the rickettsiae found in Georgia clustered with *Rickettsia* spp. identified in mosquitoes collected in 2012 in the Republic of Korea (ROK) (Maina et al., 2017). Together *R. bellii* and the rickettsiae found in mosquitoes from ROK and Georgia form a clade with clear separation from SFGR and TGR.

**TABLE 3:** Summary of metagenome next-generation sequencing and comparison of assembled rickettsial gene sequences with *Rickettsia* sp. MEAM1 (*Bemisia tabaci*) strain MEAM1 (GenBank accession CP016305).

Mosquito pool ID	Mosquito species	Number of specimens	Total number of NGS reads	Total number of rickettsial reads	Rickettsial gene	Gene coverage	Percent nucleotide identity
18KTA68A-172.44	Culex pipiens	4	208636	594	23S rRNA	56.0%	99.87%
					16S rRNA	36.0%	99.59%
18KTA68B-172.09	Culex pipiens	15	880748	7398	23S rRNA	65.0%	99.78%
18KTA68B-172.19	Culex tritaeniorhynchus	6	119378	211	23S rRNA	50.0%	99.91%
18KTA70B-178.19	Culex pipiens	10	205590	4700	23S rRNA	42.0%	97.79%
					*gltA*	82.1%	98.23%
18KTA71-179.13	Aedes caspius	20	419430	13289	23S rRNA	88.0%	99.67%
					16S rRNA	85.0%	99.46%
					*gltA*	82.1%	98.23%
18KTA71-179.39	Culex pipiens	20	706380	991	23S rRNA	61.0%	99.54%
					16S rRNA	62.0%	99.26%
18KTA71-179.40	Culex pipiens	20	802878	2043	23S rRNA	25.0%	99.13%
					16S rRNA	43.0%	94.95%
18KTA71-179.45	Culex pipiens	20	517024	439	23S rRNA	78.0%	99.37%
					16S rRNA	44.0%	99.56%
18KTA71-179.46	Culex pipiens	20	695016	35361	23S rRNA	78.0%	99.33%
					16S rRNA	48.0%	99.59%
18KTA71-179.48	Culex pipiens	20	804150	5914	23S rRNA	77.0%	99.46%
					16S rRNA	73.0%	99.52%
18KTA71-179.51	Culex pipiens	20	767540	13438	23S rRNA	70.0%	99.44%
					16S rRNA	87.0%	99.47%
18KTA71-179.55	Culex pipiens	20	918674	10320	23S rRNA	95.0%	99.49%
					16S rRNA	71.0%	99.00%
18KTA71-179.57	Culex pipiens	20	915140	558	23S rRNA	78.0%	99.77%
					16S rRNA	62.0%	99.48%
18KTA71-179.60	Culex pipiens	20	332702	22833	23S rRNA	97.0%	99.67%
					16S rRNA	91.0%	99.41%
18KTA71A-180.16	Culex pipiens	20	811394	72731	23S rRNA	96.0%	99.29%
					16S rRNA	65.0%	98.65%
18KTA71A-180.18	Culex pipiens	20	735290	8188	23S rRNA	49.0%	99.64%
					16S rRNA	38.0%	98.99%
18KTA71A-180.22	Culex pipiens	20	854426	56583	23S rRNA	92.0%	99.65%
					16S rRNA	79.0%	95.00%
18KTA71A-180.23	Culex pipiens	15	897974	3046	23S rRNA	80.0%	99.64%
18KTA71A-180.25	Culex pipiens	20	996682	15129	23S rRNA	63.0%	99.86%
18KTA71A-180.26	Culex pipiens	20	748538	118	23S rRNA	15.0%	99.69%
18KTA71A-180.27	Culex pipiens	20	767576	81	23S rRNA	27.0%	99.13%
18KTA71A-180.29	Culex pipiens	20	784246	110639	23S rRNA	79.0%	99.40%
18KTA71A-180.32	Culex pusillus	20	1148410	330756	23S rRNA	55.0%	99.56%
					16S rRNA	67.0%	97.84%
18KTA71A-180.33	Culex pusillus	20	1095486	37082	23S rRNA	83.0%	99.15%
					16S rRNA	64.0%	99.18%
18KTA74A-189.01	Aedes caspius	20	697732	32	23S rRNA	20.0%	97.88%
18KTA74A-189.03	Aedes surcoufi	4	926422	3840	23S rRNA	56.0%	99.39%
18KTA74A-189.10	Culex pusillus	16	744216	75	23S rRNA	25.0%	99.73%
18Steg 85-113.16	Aedes albopictus	1	271998	3444	23S rRNA	61.0%	99.67%
					16S rRNA	34.0%	99.43%
18Steg 86-115.01	Culex pipiens	1	199090	122	23S rRNA	59.0%	99.92%

**FIGURE 3:**
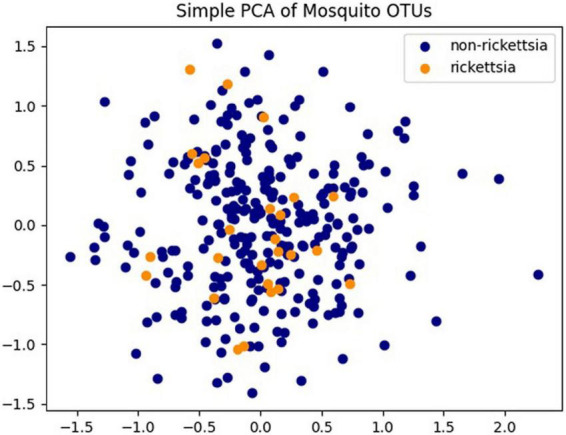
Principal component analysis (PCA) analysis of Operational Taxonomic Unit (OTU) abundances of 475 pools. Pools containing *Rickettsia* spp. are colored in orange and pools without *Rickettsia* spp. identified are colored in dark blue.

Of the 475 mosquito pools, there are 302 pools (63.6%) of *Culex* and 173 pools (36.4) of *Aedes*. Rickettsiae were found to be present in more *Culex* pools (29/33, 87.9%) than in *Aedes* pools (4/33, 12.1%). Additionally, 32/33 positive pools came from mosquitoes collected with powered traps. A single pool was positive from specimens collected *via* larval dipping and rearing them to adults prior to analysis. The 33 pools containing rickettsial sequences were from training areas KTA and NTA, with all but one found in KTA. The two training areas located in the cities of Kvemo Kartli and Norio, respectively, are both south of the capital city of Tbilisi and 30 km away from each other. Rickettsiae were not found in any other collection sites nearby. The PCA performed on all 475 pools revealed neither a distinct clustering of pools nor a distinct clustering of pools containing rickettsia species or pools not containing *Rickettsia* species (Figure 3).

To confirm the detection of rickettsiae from metagenomic sequencing and to obtain sequences of additional genes for further identification, genomic DNA was extracted from total homogenate and subjected to rickettsial qPCR, PCR amplification, and Sanger sequencing. Five samples, including Culex pools 18KTA68B-172.09 and 18KTA70B-178.19 and *Aedes* pools 18KTA70-176.01, 18KTA71-179.13, and 18Steg 85-113.16, were analyzed. Two samples, 18KTA70B-178.19 (*Culex pipiens* pool of 10 specimens) and 18KTA71-179.13 (*Aedes caspius* pool of 20 specimens), were both positive in RickCS qPCR with Ct values of 29.72 and 31.86, respectively. PCR amplification and Sanger sequencing for 16S, 23S, and *gltA* genes were also successful for these two samples. The genus specific Rick17b qPCR assay and PCR for *ompA*, *ompB*, and *sca4* genes were negative for all samples. 16S, 23S, and *gltA* sequences from Sanger sequencing were identical between the two mosquito pools and closely related to *R. bellii* (Figure 2).

## Discussion

Mosquitoes are the most abundant and most widely distributed arthropod disease vectors, transmitting more VBDs than any other insects. Although rickettsial infections occur worldwide, “hot spot” focal areas of endemicity can present a risk to travelers as well. Rickettsial diseases are difficult to diagnose and can be severe or even fatal when treatment is delayed (Stewart and Stewart, 2021). Therefore, characterizing rickettsiae in mosquitoes and other arthropods from different regions in the world offers significant medical relevance. In this study, we sequenced several conserved and variable rickettsial genes with high sequence identity to *R. bellii* from three *Culex* species and three *Aedes* species. Other reports showed *Rickettsia* spp. of different genotypes in a variety of mosquito species, including *R. felis*, which is a tick-borne human pathogen, in multiple countries (Socolovschi et al., 2012a,b; Maina et al., 2017; Zhang et al., 2019; Li et al., 2022). Thus far, there has been no clear evidence that rickettsiae are transmitted from mosquitoes to humans or animals outside of the laboratory setting, however, close attention and more investigation are needed to continue exploring the role of mosquitoes in the transmission cycle of rickettsiae and the potentiality of mosquito-borne rickettsioses.

This study detected rickettsial bacteria within multiple species of mosquito from the *Aedes* and *Culex* genera. We found one *Rickettsia* spp. positive pool from a larval dipped specimen, *Aedes albopictus* from NTA, which after the collection was allowed to emerge as an adult for identification and analysis. This result indicates that the vertical transmission of rickettsial pathogens is possible (trans-overial and trans-stadial). Our study only utilized female mosquitoes; however, previous studies have found male mosquitoes positive for *R. felis* (Zhang et al., 2016). These provide evidence that not only can rickettsia bacteria be acquired in the environment by mosquitoes but they can also be transmitted vertically.

This study showed the value of metagenomics analysis as an effective tool to support One Health efforts. The unbiased approach provides data for a comprehensive understanding of genetic contents in the study subject and possibly shed light on associations and interactions among host, viruses, other microbes, parasites, etc. Metagenomics analysis also reveals focus topics for further study. In our study, metagenome NGS detected the presence of rickettsial sequence reads in a significant number of Georgian mosquito pools, but, due to the relative abundance of rRNA (*rrs*) genes, these reads were limited to 16S and 23S rRNA genes. The results led to the application of established molecular characterization methods on selected samples to probe relatedness. It is intriguing that, in spite of the large number of NGS reads of rickettsial sequences, we were only able to succeed in relatively few rickettsial qPCR and MLST experiments. Further study is warranted to test more pools and obtain deeper sequencing data on a panel of rickettsial genes, including *rrs*, *gltA*, 17 kDa antigen, *ompB*, *ompA*, *sca4*, and *groEL* gene, to achieve taxonomic classification on the species level. We plan to use the hybridization NGS targeted enrichment method to obtain full gene sequences to complete MLST rickettsial genotyping.

We will continue the study to obtain solid evidence to further investigate the observations made in this work. Rickettsial DNA was found in both *Culex* and *Aedes* mosquitoes with *Culex* pools positive at a much higher rate than *Aedes* pools. It is noteworthy that all but one rickettsia-containing mosquito pool were from KTA, given the close proximity of KTA and NTA to each other. Future work will survey a broader diversity of mosquito species by surveying from more collection sites and conducting a statistical analysis on rickettsial carriage rates for mosquito species and the geographic distribution of mosquito-associated *Rickettsia* spp. in Georgia.

In addition to more robust and comprehensive geosurveillance and characterization studies for mosquito- associated rickettsiae, further work on the transmission and pathogenic potential is also needed. With increasing evidence on the presence of mosquito species associated with diverse rickettsial genotypes in different parts of the world, these studies are important for addressing fundamental questions on the life cycle, transmissibility, and pathogenicity of rickettsiae to humans or animals.

## Data availability statement

The original contributions presented in this study are publicly available. This data can be found here: GenBank, OP007139-OP007153, OP007301-OP007317, ON960050, and ON960051.

## Author contributions

BK, CH, SW, and DR-W: sample acquisition. AP, JJ, SL, JG, BK, TC, MC, SW, CF, DR-W, and JH: methodology and data analysis. MC, SW, CH, CF, DR-W, and JH: project administration. AP, JJ, SL, JG, BK, TC, MC, SW, CH, CF, DR-W, and JH: writing and editing. All authors contributed to the article and approved the submitted version.

## Footnotes

1
https://www.medilabsecure.com/moskeytool.html

2
https://www.geneious.com

3
https://igv.org

4
http://tree.bio.ed.ac.uk/software/figtree/

5
www.thermofisher.com/tmcalculator

## Author disclaimer

The material has been reviewed by the authors’ respective institutions. There is no objection to its presentation and/or publication. The views expressed here are those of the authors and do not reflect the official policy of the Department of the Army, Department of the Navy, Department of Defense, or the U.S. Government. This is the work of U.S. government employees and may not be copyrighted (17 USC 105).
